# Relationship Between Comprehensive Inflammatory Indicators and Gout Risk: A Cross-sectional Study Based on NHANES 2015–2018

**DOI:** 10.2174/0118715303456670260509051016

**Published:** 2026-07-27

**Authors:** Fan Zeng, Ou Mei, Min Lu, Li Wang, Gao-yan Kuang

**Affiliations:** 1 The Affiliated Hospital of Jiangxi University of Traditional Chinese Medicine, Nanchang 330006, Jiangxi, China;; 2 The First Affiliated Hospital of Hunan University of Traditional Chinese Medicine, Changsha 410007, Hunan, China

**Keywords:** Gout, inflammatory indicators, national health and nutrition examination survey, inflammation score, cross-sectional study, risk identification

## Abstract

**Introduction:**

Gout development and progression are closely linked to inflammatory processes. Six novel systemic inflammation indices, including the neutrophil-to-lymphocyte ratio (NLR), systemic immune-inflammation index (SII), inflammation score, systemic inflammation response index (SIRI), platelet-to-lymphocyte ratio (PLR), and monocyte-to-lymphocyte ratio (MLR) have shown significant value in assessing systemic inflammation severity and related comorbidities. This study aims to explore the relationship between these systemic inflammation indices and the risk of gout.

**Methods:**

Data from the 2015-2018 National Health and Nutrition Examination Survey (NHANES) were analyzed in this study to explore the link between systemic inflammation indices and gout risk. Demographic information of all participants was collected, and multivariable logistic regression was applied to evaluate the relationship between the inflammation indices and gout risk. Restricted cubic splines (RCS) were used to investigate the nonlinear association between inflammation scores and gout risk. Stratified and interaction analyses were also conducted to examine the relationships in different populations.

**Results:**

A total of 7,681 participants were included in the analysis. After adjusting for all covariates in the multivariable logistic regression model, the inflammation score demonstrated the most significant association with gout risk. Further RCS analysis revealed a nonlinear relationship (*P*=0.0134) with a turning point at 1.641. When the inflammation score was below 1.641, an increase in the score was significantly associated with a higher risk of gout. Stratified analyses indicated that age, race, and smoking status interacted with the relationship between inflammation scores and gout risk.

**Discussion:**

This study identified a significant association between the inflammation score and gout risk, particularly at low-to-moderate inflammation levels, suggesting that systemic inflammation may play an important role in the early stages of gout development. The inflammation score remained independently associated with gout after full adjustment, indicating its potential utility for early risk identification.

**Conclusion:**

The findings suggest that the inflammation score is a robust and independent predictor of gout risk, particularly at low-to-moderate levels, highlighting its potential for the early identification of high-risk individuals.

## 1 INTRODUCTION

Gout is a prevalent autoinflammatory arthritis characterized by elevated uric acid levels in the body, leading to the precipitation and deposition of monosodium urate (MSU) crystals in tissues, which trigger inflammatory responses [1]. During acute gouty arthritis attacks, patients typically experience severe joint pain accompanied by rapid swelling. Chronic and recurrent gout episodes may result in joint damage and bone erosion and, in severe cases, lead to joint dysfunction. Epidemiological studies have reported a global increase in the prevalence of gout between 1990 and 2019, with marked regional variations, with higher prevalence observed in Australasia and affluent regions of North America [2].

Therapeutic strategies for gout are generally divided into the management of acute flares and long-term urate-lowering therapy (ULT). Acute gout attacks are primarily treated with nonsteroidal anti-inflammatory drugs (NSAIDs), colchicine, or systemic corticosteroids to rapidly control inflammation and relieve symptoms, whereas interleukin-1 inhibitors may be considered in refractory cases. In contrast, long-term disease control focuses on sustained serum urate reduction to prevent recurrent flares and disease progression. Allopurinol is recommended as the first-line ULT by major clinical guidelines, while febuxostat serves as an alternative for patients who are intolerant of or inadequately responsive to allopurinol. Other urate-lowering options, including uricosuric agents such as benzbromarone and recombinant uricase for refractory gout, are also available [3]. Despite these therapeutic options, the overall clinical management of gout remains suboptimal [4]. Given the substantial impact of gout-related complications on patients’ daily lives, it is essential to further investigate the associated risk factors of gout, particularly to identify effective predictive indicators of gout risk, thereby providing a scientific basis for early clinical warning and personalized intervention.

The pathogenesis of gout is multifactorial, with systemic inflammation playing a potentially critical role in its progression [5]. Previous studies have demonstrated that alterations in peripheral blood neutrophils, lymphocytes, and specific acute-phase proteins, such as C-reactive protein, are indicative of the inflammatory responses associated with gout [6, 7]. Recently, composite inflammatory biomarkers derived from complete blood count parameters have emerged as convenient and integrated tools for assessing systemic status [8]. Indices such as the systemic inflammation response index(SIRI), neutrophil-to-lymphocyte ratio(NLR), platelet-to-lymphocyte ratio(PLR), inflammation score, and systemic immune-inflammation index(SII) havedemonstrated clinical utility in various metabolic and inflammatory conditions, including diabetes [9], cardiovascular diseases [10], rheumatoid arthritis [11], and traumatic spinal cord injuries [12]. However, despite their established relevance in multiple chronic inflammatory conditions, their association with gout remains insufficiently explored.

In this study, data from NHANES were utilized to comprehensively evaluate the associations between SIRI, PLR, SII, monocyte-to-lymphocyte ratio(MLR), inflammation score, and NLR and the risk of gout. The primary objective was to identify the most robust inflammatory biomarker associated with gout risk, thereby providing valuable insights for improved risk assessment and management of gout.

## 2 MATERIALS AND METHODS

### 2.1 Study Population

NHANES is a nationwide cross-sectional survey aimed at evaluating the health and nutritional status of the U.S. population. The study has been approved by the relevant Institutional Review Board, and its data are publicly available for free access. For this study, we utilized data from the 2015–2018 survey cycles, as only these cycles included measurements of high-sensitivity C-reactive protein (hs-CRP), a key variable required for our analysis.

### 2.2 Peripheral Inflammatory Indicators

This study included six peripheral whole blood markers: hs-CRP, white blood cell (WBC) count, neutrophil count, monocyte count, lymphocyte count, and platelet count. The calculation methods for inflammatory markers were based on previous studies. Z-scores were calculated by standardizing each biomarker to the distribution of the study population, defined as the difference between the individual value and the study population mean divided by the corresponding standard deviation. The inflammation score was derived by summing the Z-scores of hs-CRP and WBC [13]. NLR, MLR, and PLR were defined as the ratios of neutrophils, monocytes, and platelets to lymphocyte counts, respectively. Additionally, the formulas for calculating SII and SIRI were as follows: SII = (neutrophil count × platelet count) / lymphocyte count; SIRI = (neutrophil count × monocyte count) / lymphocyte count [14].

### 2.3 Definition of Gout

In this study, gout diagnosis was identified based on self-reported data from questionnaires administered during the two NHANES survey cycles (MCQ160n).

### 2.4 Covariates

This study collected various covariates to facilitate a more comprehensive analysis and interpretation of the findings. These covariates included demographic characteristics and health-related factors, specifically age, race, gender, poverty-to-income ratio, educational attainment, smoking and drinking behaviors, coronary heart disease (CHD), diabetes, and hypertension. Hypertension, CHD, and diabetes were diagnosed based on questionnaire data, while smoking status and alcohol consumption were classified using survey data. Smoking status was categorized as “smoker” or “non-smoker” based on whether participants had smoked more than 100 cigarettes in their lifetime. Alcohol intake was classified into “drinker” and “non-drinker” categories, with the threshold defined as a daily consumption of four or more drinks for women and five or more drinks for men.

### 2.5 Statistical Analysis

All following data analyses took the impact of sample weights into consideration, since NHANES used a complicated sampling design. Continuous variables were first assessed for their distributional characteristics and are presented as median (interquartile range) when non-normally distributed. Descriptive group comparisons for continuous variables were performed using the Kruskal-Wallis test, whereas categorical variables were compared using the chi-square test. The correlation between gout and composite inflammatory indicators was examined using logistic regression models. Three independent analyses were conducted, each controlling for different covariates, and trend *P*-values were computed. Model 2 included adjustments for gender, age, and race, Model 3 included adjustments for all covariates, and Model 1 was uncorrected. Also, to look for possible interactions between variables and inflammatory scores, we ran subgroup analyses. A dose-response association between inflammation scores and gout risk was then evaluated using restricted cubic spline plots and threshold effect analyses. R software (version 4.4.1) was used for statistical analysis, with a significance level of *P* < 0.05 established.

## 3 RESULTS

### 3.1 Characteristics of Participants

Initially, 19,225 participants were identified from the NHANES 2015-2018 cycles. After excluding individuals younger than 18 years, those with missing gout-related information, and those with incomplete covariate data, a final analytic sample of 7,681 participants remained, 426 of whom were diagnosed with gout (Fig. **1**).

### 3.2 Baseline Characteristics of Participants

Baseline characteristics showed that, compared with participants without gout, those with gout were significantly older and predominantly male. Significant differences were also observed in the prevalence of diabetes and hypertension, as well as in smoking history and alcohol consumption. Furthermore, participants with gout exhibited elevated inflammation scores and higher levels of various inflammatory markers, including hs-CRP, WBC, lymphocyte count, monocyte count, platelet count, and neutrophil count, as well as NLR, MLR, and SIRI (Table **1**).

### 3.3 Association of Peripheral Inflammation Indicators with Gout Risk

The results of the multivariable logistic regression analysis are presented in Table **2**, demonstrating the relationships between peripheral blood inflammatory markers, composite inflammatory indices, and the risk of gout. Among the various peripheral blood inflammatory markers, neutrophils showed a statistically significant association with gout risk in both Model 1 and Model 2, whereas the correlations between lymphocytes, monocytes, and platelets with gout risk were largely non-significant. In contrast, hs-CRP exhibited the most pronounced association with gout risk, and this association remained consistent and robust across Models 1, 2, and 3.

Model 1 (unadjusted model) revealed a significant positive association between the inflammatory score and gout risk (OR=1.19, 95% CI 1.10–1.28; *P*<0.001). In Model 2, after adjusting for selected demographic variables (age, gender, and race), the positive association remained consistent (OR=1.19, 95% CI 1.09–1.30; *P*<0.001) with statistical significance. Model 3, which further adjusted for all remaining covariates, showed that the positive association between the inflammatory score and gout risk remained stable (OR=1.13, 95% CI 1.02–1.24; *P*=0.018). In addition, SIRI showed a statistically significant association with gout risk in Models 1 and 2 but lost significance in Model 3. Conversely, NLR, MLR, PLR, and SII did not exhibit statistically significant associations with gout risk in Models 2 and 3. Overall, the inflammatory score demonstrated the most robust and consistent association with gout risk across all models.

Therefore, this study further explored the nonlinear relationship between inflammation scores and gout risk using RCS functions. The results indicated a significant nonlinear association between inflammation scores and gout risk (Fig. **2**). Threshold effect analysis identified a turning point at an inflammation score of 1.641, demonstrating differing regression slopes before and after the turning point (Table **3**). When the inflammation score was below 1.641, gout risk significantly increased with each unit rise in the inflammation score, corresponding to a 52.4% higher risk. However, above this threshold, no significant association between inflammation scores and gout risk was observed.

### 3.4 Inflammation Score-gout Relationship Subgroup Analysis

Subgroup and interaction analyses were performed to examine whether the association between inflammation scores and gout risk varied across different populations. Participants were stratified according to gender, race, age, educational attainment, smoking habits, alcohol intake, hypertension, diabetes, and CHD. The results are presented in Fig. (**3**). Statistically significant associations between inflammation score and gout risk were observed in the following subgroups: individuals aged <60 years, males, Mexican Americans, Other Hispanics, Non-Hispanic Whites, Less Than 9th Grade, Some College or AA degree, drinkers, smokers, individuals without hypertension, and those with diabetes. Interaction tests revealed that age, race, and smoking status significantly influenced the association between inflammation score and gout risk (Fig. **3**).

## 4 DISCUSSION

This study represents the first cross-sectional investigation into the relationship between composite inflammatory markers and gout risk, utilizing data from two survey cycles (2015–2018) of the NHANES database. Multiple analytical methods were employed to comprehensively evaluate the association between composite inflammatory markers and gout. Among the six composite inflammatory markers examined, only the inflammation score showed a significant association with gout risk. Participants with gout exhibited significantly higher inflammation scores than those without gout. Furthermore, after adjustment for all covariates, the inflammation score remained the most robust and independently associated marker with gout risk.

Interaction analysis demonstrated significant differences in the relationship between the inflammation score and gout risk across different subgroups, including age, race, and smoking status. Notably, RCS analysis identified a non-linear association with a critical turning point at 1.641. From a clinical perspective, this cutoff of 1.641 might offer a useful benchmark for risk stratification. Below this value, the positive association between systemic inflammation and gout risk appears particularly pronounced, representing a 'rapid escalation zone'. For clinicians, this implies that individuals with an inflammatory score approaching 1.641--even if their individual markers (such as WBC count or hs-CRP) are still within the 'high-normal' range--should be identified as high-risk candidates for gout. Monitoring this composite score may provide a more sensitive early warning than assessing single inflammatory markers in isolation, allowing for a 'window of opportunity' for primary prevention through anti-inflammatory lifestyle interventions or dietary modifications. Once the inflammation score exceeds 1.641, the association with gout risk reaches a plateau and becomes non-significant. This saturation effect could suggest that inflammation might play a more critical role as a 'trigger' in the early stages of gout onset. Low-to-moderate levels of inflammation could act as a 'trigger phase,' leading to rapid uric acid accumulation or increased inflammatory factors. However, the subsequent leveling off may be ascribed to the initiation of compensatory anti-inflammatory reactions. As previous studies indicated, while early-to-middle phases of inflammation involve a marked increase in inflammatory cells inside synovial fluid, the latter stages may see an elevation in anti-inflammatory substances and the generation of neutrophil extracellular traps (NETs), which can destroy inflammatory molecules and alleviate gout-related inflammation [15].

Furthermore, other composite inflammatory markers analyzed in this study, including NLR, MLR, PLR, SII, and SIRI, exhibited weaker associations with gout risk and were more susceptible to covariate adjustment. In contrast, the inflammation score demonstrated superior robustness and a stronger association with gout among the six markers, suggesting its potential utility as an early risk indicator for gout.

Gout is a common joint disorder, and previous studies have demonstrated that factors such as immune-mediated inflammatory mechanisms, age, and sex are closely associated with its development [16,17]. Consistent with these findings, our study also shows that men are at a significantly higher risk of gout than women, that individuals with gout tend to be older, and exhibit higher overall levels of systemic inflammation compared with those without gout. As research into gout-related mechanisms has advanced,, the inflammatory activation pathways have been further elucidated. Studies have shown that immune cells, including neutrophils, monocytes, and lymphocytes, play crucial roles in the pathogenesis of gout [18]. Neutrophils, the predominant subgroup of white blood cells, are primarily recruited to inflamed tissues during the acute phase of inflammation to combat pathogens [19]. During the progression of gout, neutrophils phagocytose MSU crystals, triggering a massive release of inflammatory mediators, which constitutes a critical process in acute gouty inflammation [20]. Additionally, CRP is an essential biomarker of acute-phase inflammation. In response to tissue injury or inflammatory stimuli, CRP levels rise rapidly, subsequently interacting with platelets and white blood cells and thereby modulating systemic inflammatory responses [21]. Recent studies indicate that CRP serves as a key biomarker for both acute and chronic inflammatory disorders and is strongly linked to conditions like gout [22–24]. Evidence suggests that patients with hyperuricemia exhibit significantly higher levels of CRP and white blood cells compared to healthy individuals [25]. In addition, elevated CRP levels have been linked to an increased risk of all-cause mortality in patients with gout [26]. However, the elevation of single inflammatory markers may be influenced by other factors. Therefore, combined inflammatory markers offer a broader evaluation of the body's overall inflammatory condition.

Composite inflammatory indicators derived from peripheral blood were initially developed to assess the intensity of systemic inflammation. Subsequent studies have demonstrated their significant predictive utility across various inflammatory pathologies. Due to their ease of accessibility, low cost, and high reproducibility, these markers have garnered widespread attention from clinicians [27]. Composite inflammatory markers based on absolute counts of lymphocytes, platelets, and neutrophils have been extensively utilized for disease prognosis, recurrence monitoring, and evaluation of therapeutic efficacy [28]. Representative indices include NLR, MLR, PLR, SII, SIRI, and the inflammation score, all of which have shown significant value in assessing systemic inflammation severity and related comorbidities [29, 30]. NLR is a key marker for evaluating systemic infection risk and inflammatory status, effectively predicting the prognosis of stroke patients [31]. and mortality in community-acquired pneumonia [32]. MLR is closely associated with cardiovascular diseases and serves as an inflammatory marker linked to increased cardiovascular risk [33]. PLR, derived from platelet and lymphocyte counts, effectively reflects systemic inflammatory responses and thrombotic activity, and has been reported as a potential predictor of acute coronary syndrome [34]. SII, as a novel biomarker, has shown strong correlations with malignancies and inflammation-related diseases, and has been reported to predict the development of vasospasm during the progression of aneurysmal subarachnoid hemorrhage (SAH) [35]. SIRI reflects the interaction between immune status and inflammatory responses, and evidence suggests that it may be useful in predicting early postoperative recurrence in hepatocellular carcinoma [36]. In addition, a bibliometric analysis combined with a large-scale retrospective study evaluating inflammatory markers, including NLR, MLR, SIRI, and PLR, in patients with osteoarthritis and healthy controls demonstrated a significant association between SIRI and osteoarthritis disease activity [37]. The aforementioned composite inflammatory markers are calculated based on leukocyte subtypes but do not include critical inflammatory indicators such as CRP. In contrast, the inflammation score is a novel composite marker designed to assess systemic inflammatory burden, typically calculated as the sum of Z-scores for WBC and hs-CRP. This approach combines hs-CRP and WBC, two key inflammatory markers, offering a broader and more systematic representation of the body's inflammatory condition [38]. Therefore, exploring the relationship between the inflammation score and gout risk is of substantial clinical value for optimizing treatment strategies and improving the prognosis of gout patients.

This study demonstrates a significant and consistent association between the inflammatory score and an increased risk of gout, particularly at low to moderate levels of systemic inflammation, where the risk of gout shows a marked increase. These findings highlight a potential window for early intervention, suggesting that individuals with scores in this range may benefit most from proactive lifestyle modifications, such as targeted dietary management and physical activity. Implementing such measures could facilitate more precise risk stratification in clinical practice and enable timely inflammatory management, potentially reducing the incidence of gout and its associated complications. However, it remains unclear whether the inflammation score partially reflects gout severity, requiring additional research in the future.

## 5 LIMITATIONS

Despite the significant association observed between the inflammation score and gout risk, several limitations should be acknowledged. First, although multiple demographic and clinical covariates were adjusted for, residual confounding from unmeasured factors such as dietary habits, body mass index, and medication use cannot be excluded. Second, gout status was identified based on self-reported questionnaire data in NHANES, which may have introduced recall bias or misclassification. Third, due to data availability constraints, hs-CRP measurements were limited to the 2015–2018 survey cycles, potentially reducing statistical power. In addition, while participants with severe acute infections are unlikely to be included, mild or asymptomatic infections could not be fully excluded and may have influenced systemic inflammatory markers. Finally, owing to the cross-sectional design, causal relationships between inflammation score and gout cannot be established, and prospective studies are warranted.

## CONCLUSION

Compared to single inflammatory indicators, composite inflammatory indices can more comprehensively reflect both local and systemic immune-inflammatory responses, demonstrating higher diagnostic and prognostic value in various clinical settings. This study preliminarily explored the role of NLR, MLR, PLR, SII, SIRI, and the inflammatory score in assessing gout risk. The results showed that the association between the inflammatory score and gout risk was the most significant and robust. Despite certain limitations, this cross-sectional study provides a relatively comprehensive analysis, highlighting the potential value of the inflammatory score as a predictive tool for identifying high-risk gout patients, with important clinical implications.

## Figures and Tables

**Fig. (1):**
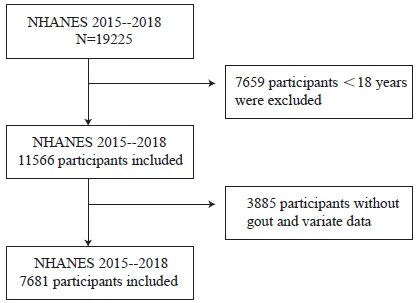
Flowchart of participant selection.

**Fig. (2):**
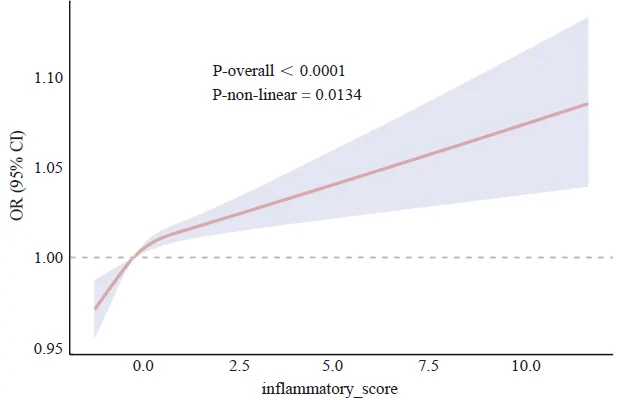
Smooth curve fitting for inflammation score and gout. **Note:** The analysis included 7,681 participants (426 with gout, 7,255 without). The solid line and shaded area represent the estimated odds ratios (ORs) and 95% confidence intervals (CIs), respectively. The curve was fitted using multivariable logistic regression adjusted for all covariates. P-non-linear = 0.0134 indicates a significant nonlinear dose-response relationship.

**Fig. (3):**
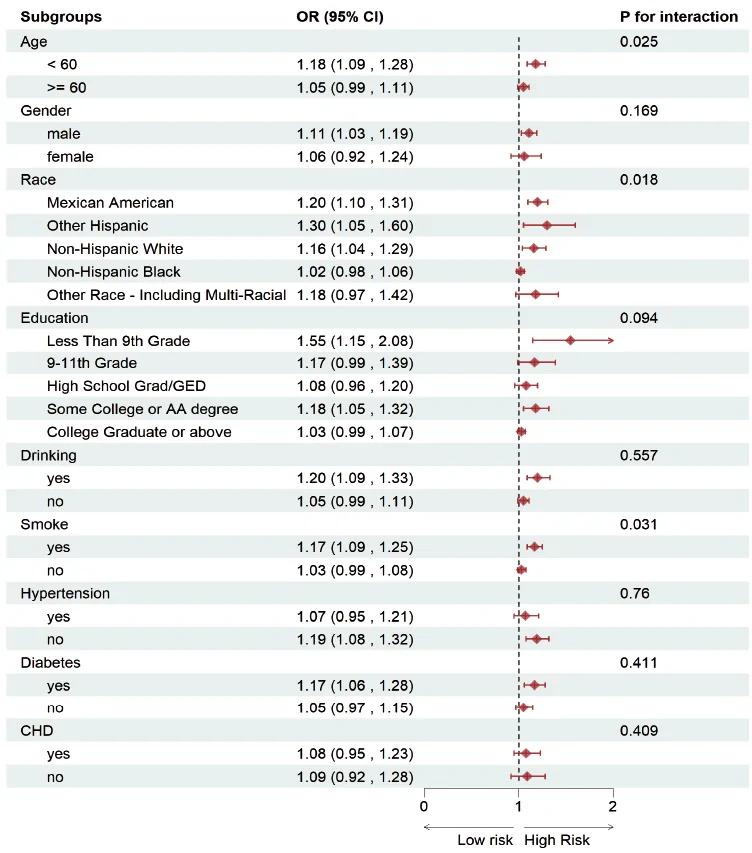
Subgroup analysis for the association between inflammation score and gout. **Note:** The forest plot displays odds ratios (ORs) and 95% confidence intervals (CIs) across different subpopulations (Total N = 7,681). Participants were stratified by age, gender, race, education, smoking, drinking, hypertension, diabetes, and CHD. ORs were calculated using logistic regression models. The “P for interaction” denotes the statistical significance of the interaction between stratification variables and the inflammation score.

**Table 1:** Baseline characteristics of participants.

-	**Gout**		-	
**Characteristic**	**Yes** N = 426	**No** N = 7255	**Overall** N = 7681	***P-value***
Age (years)	62.00(55.00, 70.00)	47.00(33.00, 60.00)	48.00 (33.00, 61.00)	<0.001
Sex, n(%)	-	-	-	<0.001
female	116 (30.00)	3579 (51.00)	3695 (50.00)	-
male	310 (70.00)	3676 (49.00)	3986 (50.00)	-
Race, n(%)	-	-	-	0.046
Non-Hispanic White	175 (70.00)	2722 (66.00)	2897 (67.00)	-
Non-Hispanic Black	108 (12.00)	1539 (10.00)	1647 (10.00)	-
Mexican American	37 (4.60)	1149 (8.80)	1186 (8.60)	-
Other Race- Including Multi-Racial	70 (9.80)	1043 (8.40)	1113 (8.50)	-
Other Hispanic	36 (4.40)	802 (6.40)	838 (6.30)	-
Diabetes, n(%)	-	-	-	<0.001
Yes	158 (27.00)	1044 (11.00)	1202 (11.00)	-
No	268 (73.00)	6211 (89.00)	6479 (89.00)	-
CHD, n(%)	-	-	-	<0.001
Yes	62 (15.00)	301 (3.50)	363 (4.00)	-
No	364 (85.00)	6954 (96.00)	7318 (96.00)	-
Hypertension, n(%)	-	-	-	<0.001
Yes	300 (68.00)	2536 (30.00)	2836 (32.00)	-
No	126 (32.00)	4719 (70.00)	4845 (68.00)	-
Education levels, n(%)	-	-	-	0.5
Less Than 9th Grade	35 (3.60)	582 (3.60)	617 (3.60)	-
9-11th Grade	50 (7.60)	784 (7.40)	834 (7.40)	-
High School Grad/GED	101 (25.00)	1717 (24.00)	1818 (24.00)	-
Some College or AA degree	153 (37.00)	2362 (32.00)	2515 (33.00)	-
College Graduate or above	87 (27.00)	1810 (32.00)	1897 (32.00)	-
Smoking, n(%)	-	-	-	<0.001
Yes	271 (63.00)	3409 (47.00)	3680 (47.00)	-
No	155 (37.00)	3846 (53.00)	4001 (53.00)	-
Drinking, n(%)	-	-	-	<0.001
Yes	117 (26.00)	1157 (15.00)	1274 (16.00)	-
No	309 (74.00)	6098 (85.00)	6407 (84.00)	-
Inflammatory score	-0.14 (-0.52, 0.42)	-0.29 (-0.64, 0.21)	-0.28 (-0.63, 0.22)	0.002
hs-CRP (mg/L)	2.60 (1.10, 6.20)	1.80 (0.80, 4.30)	1.80 (0.80, 4.40)	<0.001
WBC (10^9^/L)	7.40 (6.30, 8.80)	7.00 (5.80, 8.60)	7.00 (5.90, 8.60)	0.045
Lymphocytes (10^9^/L)	2.00 (1.50, 2.50)	2.10 (1.70, 2.60)	2.10 (1.70, 2.60)	0.035
Monocytes (10^9^/L)	0.60 (0.50, 0.70)	0.60 (0.50, 0.70)	0.60 (0.50, 0.70)	<0.001
PLT (10^9^/L)	217.00 (172.00, 263.00)	236.00 (202.00, 274.00)	235.00 (202.00, 274.00)	<0.001
Neutrophils (10^9^/L)	4.30 (3.30, 5.40)	4.10 (3.20, 5.20)	4.10 (3.20, 5.20)	0.044
NLR	2.14 (1.56, 2.93)	1.94 (1.48, 2.57)	1.95 (1.48, 2.59)	0.003
MLR	0.31 (0.25, 0.40)	0.27 (0.21, 0.33)	0.27 (0.21, 0.33)	<0.001
PLR	110.00 (83.00, 147.00)	114.00 (90.00, 141.00)	113.00 (90.00, 142.00)	0.4
SII	478.00 (313.00, 705.00)	459.00 (330.00, 637.00)	460.00 (329.00, 640.00)	0.5
SIRI	1.26 (0.90, 2.04)	1.09 (0.75, 1.60)	1.10 (0.76, 1.61)	<0.001

**Note:** Dates are presented as median (IQR) or n (%). **Abbreviations:** CHD, coronary heart disease; hs-CRP, high-sensitivity C-reactive protein; PLT, platelet count; WBC, white blood cell; NLR, neutrophil-to-lymphocyte ratio; MLR, monocyte-to-lymphocyte ratio; PLR, platelet-to-lymphocyte ratio; SII, systemic immune-inflammation index; SIRI, systemic inflammation response index; LBDLYMNO, lymphocyte count; LBDMONO, monocyte count; LBDNENO, neutrophil count; LBXPLTSI, platelet count.

**Table 2:** The association of the inflammatory score and other inflammatory markers with gout.

**Variables**	**OR**	**95% CI**	***P*-value **
**hs-CRP**	-	-	-
Model 1	1.03	1.02,1.04	<0.001
Model 2	1.03	1.02,1.04	<0.001
Model 3	1.02	1.01,1.04	0.002
**LBDLYMNO**	-	-	-
Model 1	0.97	0.77,1.24	0.8
Model 2	1.00	0.98,1.02	>0.9
Model 3	0.99	0.95,1.03	0.7
**LBDMONO**	-	-	-
Model 1	2.70	1.72,4.24	<0.001
Model 2	1.53	0.89,2.63	0.12
Model 3	0.94	0.52,1.70	0.8
**LBXPLTSI**	-	-	-
Model 1	1.00	0.99,1.00	0.009
Model 2	1.00	1.00,1.00	>0.9
Model 3	1.00	1.00,1.00	0.8
**LBDNENO**	-	-	-
Model 1	1.10	1.03,1.17	0.009
Model 2	1.13	1.05,1.21	0.002
Model 3	1.07	0.99,1.15	0.086
**Inflammatory score**	-	-	-
Model 1	1.19	1.10,1.28	<0.001
Model 2	1.19	1.09,1.30	<0.001
Model 3	1.13	1.02, 1.24	0.018
**NLR**	-	-	-
Model 1	1.18	1.09,1.28	<0.001
Model 2	1.08	0.99,1.16	0.070
Model 3	1.05	0.96,1.15	0.2
**MLR**	-	-	-
Model 1	8.19	4.61,14.5	<0.001
Model 2	1.10	0.51,2.40	0.8
Model 3	0.83	0.32,2.17	0.7
**PLR**	-	-	-
Model 1	1.00	1.00,1.00	0.7
Model 2	1.00	1.00,1.00	0.3
Model 3	1.00	1.00,1.00	0.5
**SII**	-	-	-
Model 1	1.00	1.00,1.00	0.061
Model 2	1.00	1.00,1.00	0.14
Model 3	1.00	1.00,1.00	0.3
**SIRI**	-	-	-
Model 1	1.30	1.21,1.40	<0.001
Model 2	1.13	1.03,1.25	0.014
Model 3	1.06	0.94,1.19	0.3

**Note:** Model 1: Unadjusted; Model 2: Adjusted for age, gender, and race; Model 3: Further adjusted for all covariates; OR, Odds ratio; CI, Confidence interval. **Abbreviations:** hs-CRP, high-sensitivity C-reactive protein; NLR, neutrophil-to-lymphocyte ratio; MLR, monocyte-to-lymphocyte ratio; PLR, platelet-to-lymphocyte ratio; SII, systemic immune-inflammation index; SIRI, systemic inflammation response index; LBDLYMNO, lymphocyte count; LBDMONO, monocyte count; LBDNENO, neutrophil count; LBXPLTSI, platelet count.

**Table 3:** Analysis of threshold effects.

-	-	Adjusted OR(95%CI)	*P*-value
Fitting model by standard linear regression	inflammation score	1.085(1.033-1.148)	0.004
Fitting model by two-piecewise linear regression	-	-	-
-	Inflection point	1.641	-
-	<1.641	1.524(1.318-1.76)	0
-	>1.641	1.011(0.944-1.06)	0.687

**Note:** The table compares a standard linear regression model with a two-piecewise linear regression model to identify the inflection point. Adjusted ORs and 95% CIs were calculated by adjusting for all covariates (age, gender, race, education levels, smoking, drinking, hypertension, diabetes, and CHD) and incorporating complex sample weights.

## Data Availability

The data sets used and/or analysed during this study are available from the corresponding author upon request.

## LIST OF ABBREVIATIONS

NLR: Neutrophil-to-lymphocyte Ratio
MLR: Monocyte-to-lymphocyte Ratio
PLR: Platelet-to-lymphocyte Ratio
SII: Systemic Immune-inflammation Index
SIRI: Systemic Inflammation Response Index
NHANES: National Health and Nutrition Examination Survey
RCS: Restricted Cubic Splines
NSAIDs: Nonsteroidal Anti-inflammatory Drugs
hs-CRP: High-sensitivity C-reactive Protein
WBC: White Blood Cell
CHD: Coronary Heart Disease

## STANDARDS OF REPORTING

STROBE guidelines and methodologies were followed.
